# Thioarsenate Formation Coupled with Anaerobic Arsenite Oxidation by a Sulfate-Reducing Bacterium Isolated from a Hot Spring

**DOI:** 10.3389/fmicb.2017.01336

**Published:** 2017-07-14

**Authors:** Geng Wu, Liuqin Huang, Hongchen Jiang, Yue’e Peng, Wei Guo, Ziyu Chen, Weiyu She, Qinghai Guo, Hailiang Dong

**Affiliations:** ^1^State Key Laboratory of Biogeology and Environmental Geology, China University of Geosciences Wuhan, China; ^2^State Key Laboratory of Biogeology and Environmental Geology, China University of Geosciences Beijing, China; ^3^Department of Geology and Environmental Earth Science, Miami University, Oxford OH, United States

**Keywords:** thioarsenate, hot springs, anaerobic arsenite oxidation, *arxA* gene, sulfate-reducing bacterium

## Abstract

Thioarsenates are common arsenic species in sulfidic geothermal waters, yet little is known about their biogeochemical traits. In the present study, a novel sulfate-reducing bacterial strain *Desulfotomaculum* TC-1 was isolated from a sulfidic hot spring in Tengchong geothermal area, Yunnan Province, China. The *arxA* gene, encoding anaerobic arsenite oxidase, was successfully amplified from the genome of strain TC-1, indicating it has a potential ability to oxidize arsenite under anaerobic condition. In anaerobic arsenite oxidation experiments inoculated with strain TC-1, a small amount of arsenate was detected in the beginning but became undetectable over longer time. Thioarsenates (AsO_4-x_S_x_^2-^ with *x* = 1–4) formed with mono-, di- and tri-thioarsenates being dominant forms. Tetrathioarsenate was only detectable at the end of the experiment. These results suggest that thermophilic microbes might be involved in the formation of thioarsenates and provide a possible explanation for the widespread distribution of thioarsenates in terrestrial geothermal environments.

## Introduction

High concentrations of arsenic have been reported in global terrestrial hot springs (McKenzie et al., 2001; Cleverley et al., 2003; Aiuppa et al., 2006; Pascua et al., 2007; Landrum et al., 2009; McCleskey et al., 2010). Traditionally, the predominant form of inorganic arsenic in aqueous environments is arsenate [As(V) as H_2_AsO_4_^-^ and HAsO_4_^2-^] and arsenite [As(III) as H_3_AsO_3_^0^ and H_2_AsO_3_^-^] in oxic and anoxic environments, respectively (Oremland, 2003). However, recently pentavalent arsenic-sulfur species, so-called thioarsenates (AsO_4-x_S_x_^2-^ with *x* = 1–4), have also been reported as important arsenic species in a number of sulfidic geothermal environments (Wilkin et al., 2003; Stauder et al., 2005; Planer-Friedrich et al., 2007, 2009; Härtig and Planer-Friedrich, 2012; Hug et al., 2014). For example, Hug et al. (2014) found that di- (*x* = 2) and tri-thioarsenates (*x* = 3) represented up to 25% of total arsenic in an acidic-sulfidic hot spring in New Zealand. Keller et al. (2014) investigated the arsenic speciation in natural alkaline-sulfidic geothermal waters (pH 8.56–9.60) and found that sulfide concentration and pH are the predominant factors determining the arsenic species distribution.

Extensive studies have shown that microbial activities can strongly influence the speciation and mobility of arsenic in natural environments through arsenic oxidation and reduction (Oremland et al., 2005; Paez-Espino et al., 2009). Most (if not all) of known arsenite-oxidizing microorganisms contain arsenite oxidases, which catalyze the transformation of arsenite [As(III)] to arsenate [As(V)] (Lett et al., 2012). The arsenite oxidases are encoded by *aioA* and *arxA* genes for aerobic and anaerobic arsenite-oxidizing bacteria, respectively. Thus the *aioA* and *arxA* genes have become molecular biomarkers to study the distribution and activity of arsenite-oxidizing bacteria in natural environments (Hamamura et al., 2009, 2010, 2014; Zargar et al., 2012; Engel et al., 2013; Jiang et al., 2014; Wu et al., 2015; Hernandez-Maldonado et al., 2016). Recently, it is speculated that filamentous microbial mats might play an important role in thioarsenate transformation in an alkaline, sulfidic hot spring in Yellowstone National Park, which is the first evidence showing microbially mediated thioarsenate species transformation by (hyper) thermophilic prokaryotes (Härtig and Planer-Friedrich, 2012). A subsequent investigation showed that the thermophilic microbial mats were mainly composed of *Aquificales* represented by *Thermocrinis* spp. and *Sulfurihydrogenibium* spp. (Planer-Friedrich et al., 2015). However, little is known about which microbial group was involved in the observed thioarsenate species transformation. In addition, one geochemical study on arsenic speciation in the Tengchong geothermal zone (TGZ) of Yunnan Province, China reported that thioarsenates are widely distributed in the high-sulfidic Tengchong hot springs (Guo et al., 2017). Thus, the TGZ hot springs are suitable sites for retrieving microorganisms potentially involved in thioarsenates transformation.

In the present study, we provided biological evidence on the potential involvement of a novel sulfate-reducing bacterium isolated from a TGZ hot spring, designated as *Desulfotomaculum* sp. TC-1, in the formation of thioarsenates. The strain TC-1 cells coupled sulfate reduction with arsenite oxidation, in which thioarsenates, instead of arsenate, were the main products. The results in this study suggested that microbial activities may be involved in the formation of thioarsenates in geothermal features and thus could explain the reported distribution of thioarsenates in sulfidic hot springs.

## Materials and Methods

### Site Description and Sample Collection

The TGZ is located at the collision boundary between the Indian and Eurasian plates. The TGZ is known for its various geothermal features, which contains more than 800 hot springs (Du et al., 2005; Guo and Wang, 2012). Previous studies have shown that Tengchong hot springs host very diverse microbial communities (Hedlund et al., 2012; Hou et al., 2013; Song et al., 2013a; Briggs et al., 2014), which play important roles in the elemental cycling (e.g., carbon, nitrogen, sulfur) and arsenic transformation (Jiang et al., 2010, 2014; Song et al., 2013b; Li et al., 2015; Wu et al., 2015; Yang et al., 2015; Chen et al., 2016). A hot spring (N24.95318°; E98.43838°) was found downstream of the Dagunguo (DGG) spring in the Rehai Geothermal National Park in the TGZ and was therefore named Dagunguo-2 (DGG-2) (**Supplementary Figure S1**) (Guo et al., 2017). In June 2014, water temperature and pH were measured in the field with a portable meter (LaMotte, Chestertown, MD, United States). Water chemistry (S^2-^, Fe^2+^, NO_2_^-^ and NH_4_^+^) measurements were performed by using Hach kits (Hach Company, Loveland, CO, United States). Sediment samples of the sampled hot spring were aseptically collected for cultivation.

### Enrichment and Isolation of Thermophilic Sulfate Reducing Bacteria

Hungate techniques were used for enrichment and isolation. The hot spring sediment samples were transferred into 25 mL Balch tubes containing 5 mL DSMZ medium 63, which was pre-prepared anaerobically with the headspace filled with 100% N_2_ gas. *In situ* enrichments (by putting the culture tubes in the hot spring) were incubated for 48 h and then the resulting enrichment cultures were transported to laboratory for further isolation and purification. To avoid light effects, the balch tubes were covered with foil *in situ* and *ex situ* during incubation. Inoculation, sampling, and isolation were performed in an anaerobic glove box (COY Laboratory Products, Grass Lake, MI, United States) with aseptic techniques (The gas chamber was filled with 100% N_2_). The incubation temperature was 60°C. Cultures with positive growth (as indicated by the formation of black ferrous iron sulfide) were transferred three times for further purification. Isolation was performed by using the rolling-tube method with a high-melting-point agar, GELRITE gellan gum (Sigma) (Hungate and Macy, 1973).

### SEM Observation

The morphology of strain TC-1 cells was examined with a Zeiss Supra 55 SAPPHIRE scanning electron microscope (SEM) using 7–10 keV accelerating voltage and 8.5 mm working distance. SEM sample preparation and observation were performed according to previously described methods (Zhao et al., 2013, 2015).

### Phylogenetic Analysis of Strain TC-1

Total DNA of strain TC-1 was extracted with Bacterial DNA extraction kit (ABigen, Hangzhou, China) according to manufacturer’s protocol. The 16S rRNA and *arxA* genes were amplified with the primer sets of Bac27F/Univ1492R and arxA_Deg_F_B (5′-CCA TCW SCT GGR ACG AGG CCY TSG-3′)/arxA_Deg_R_B (5′-GTW GTT GTA GGG GCG GAA S-3′) (Zargar et al., 2012), respectively. PCR amplification, sequencing, and phylogenetic analysis of the 16S rRNA and *arxA* genes were performed as previously described (Weisburg et al., 1991; Zargar et al., 2010, 2012). The *arxA* and 16S rRNA gene sequences of strain TC-1 were deposited in the GenBank under accession numbers of KX242336 and KX242337, respectively.

### Test for Arsenite Oxidation of Strain TC-1

Strain TC-1 was grown at 60°C in anoxic DSMZ 63 medium (1 L) made of solution A [K_2_HPO_4_ 0.5 g; NH_4_Cl,1.0 g; Na_2_SO_4_,1.0 g; CaCl_2_ × 2 H_2_O, 0.1 g; MgSO_4_ × 7 H_2_O, 2.0 g; Na-DL-lactate, 2.0 g; yeast extract, 1.0 g; Na-resazurin solution (0.1% w/v), 0.5 ml; distilled water, 980.0 ml], solution B (FeSO_4_ × 7 H_2_O, 0.5 g; Distilled water, 10.0 ml) and solution C (ascorbic acid, 0.1 g; distilled water, 10.0 ml). The initial pH was 6.8, and pH varied less than 0.2 units during growth. To remove oxygen, the growth medium was boiled for >10 min, purged with N_2_ upon cooling and immediately transferred into an anoxic chamber (100% N_2_) for dispensing into glass serum bottles, which were subsequently sealed with thick butyl rubber stoppers. The sets of experiments were inoculated with TC-1 in DSMZ 63 medium with solution B replaced by 0.5 mM arsenite; Two types of abiotic controls were set up: one was in the DSMZ 63 medium with solution B replaced by 5 mM sulfide (Na_2_S⋅9 H_2_O, Sigma–Aldrich) and 0.5 mM arsenite (Sigma–Aldrich), and the other was in the DSMZ 63 medium with solution B replaced by 5 mM sulfide and 0.5 mM arsenate (Sigma–Aldrich). All experimental treatments were performed in triplicate. Liquid sampling for arsenic measurement was performed with aseptic syringes in an anaerobic chamber according to a previous method (Planer-Friedrich et al., 2015).

### Arsenic Speciation Measurement for Total Arsenic, As (III) and As(V)

Total arsenic concentration and arsenic speciation (arsenite and arsenate) were measured according to previously described methods (Wu et al., 2015). Briefly, total arsenic concentration was measured with inductively coupled plasma atomic emission spectroscopy (ICP-AES) (iCAP ICP Spectrometer, Thermo Fisher Scientific, United States) with argon torch and iTeva software (Thermo Fisher Scientific, United States). Arsenic speciation (arsenite and arsenate) was determined with high performance liquid chromatography (HPLC)-atomic fluorescence spectroscopy (AFS). If the sum of measured As(III) and As (V) was not equal to total arsenic, those samples were oxidized by H_2_O_2_, in which As(III) and Thio-arsenate will be transferred to As(V) (Pettine et al., 1999), and then were measured for total As by ICP-AES.

### Characterization of Thioarsenic Species with Liquid Chromatography-High Resolution Mass Spectrometry (LC-HRMS)

The relative abundances of four major thioarsenic species in the samples, including H_3_AsSO_3_, H_3_AsS_2_O_2_, H_3_AsS_3_O and H_3_AsS_4_, were identified by injecting a liquid sample (5 μL) into a liquid chromatography-high resolution mass spectrometry (LC-HRMS, Q Exactive, Thermo Scientific, Germany) according to previously described methods (Chang et al., 2015; Yang et al., 2016). The mobile phase contained 50% acetonitrile (v/v) and 0.1% acetic acid (m/v) with a flow rate of 0.25 mL min^-1^. The mass spectrometer system was operated with a heated electrospray ionization (HESI) source in a negative ion mode with a spray voltage of -3.2 kV, an S-lens RF level of 50%, a capillary temperature of 300°C, and a mass resolution of 70,000. The runtime was 2 min for each sample. The mass tolerance of the Precursor ion was below 5 ppm. Mass spectra were processed by using the Xcalibur 2.1 software (Thermo Scientific). The relative abundance of each thioarsenic species was calculated according to their corresponding chromatographic peak area.

## Results

### Water Chemistry of the Dagunguo-2 Hot Spring

The pH and temperature of the Dagunguo-2 hot spring were 5.5 and 58.3°C, respectively. The spring water contained S^2-^ (1.53 μM), Fe^2+^ (5 μM), NO_2_^-^ (0.065 μM), and NH_4_^+^ (37.8 μM).

### Isolate Identification and Physiological Characterization

**FIGURE 1:**
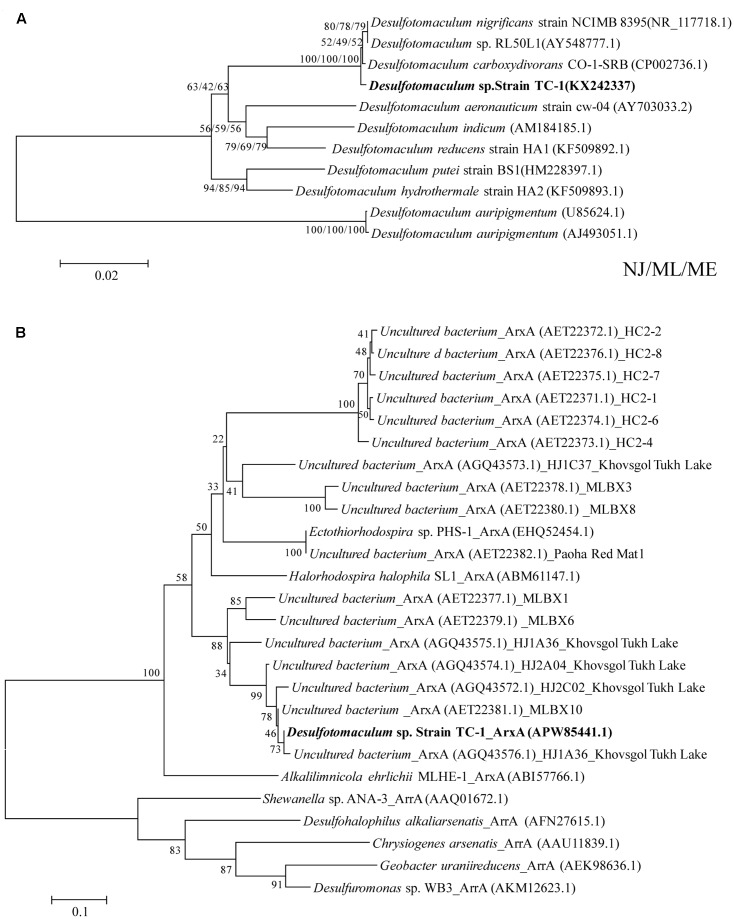
Phylogenetic trees of 16S rRNA **(A)** and the deduced amino acid sequences of *ArxA* **(B)** encoded by *arxA* genes of strain TC-1 showing their relatedness to its close relatives in the GenBank. The GenBank accession numbers are listed in parentheses. Bootstrap values (per 1000 trials) > 50% are indicated. NJ/ML/ME indicates Neighbor-Joining/Maximum Likelihood/Minimum-Evolution algorithms.

One strain was obtained and designated as strain TC-1 (**Supplementary Figure S2**). Phylogenetic analysis on the basis of 16S rRNA gene sequence identified strain TC-1as a close (sequence identity: 99.7%) relative of a sulfate-reducing bacterium *Desulfotomaculum carboxydivorans* CO-1-SRB, which was isolated from a sludge of an anaerobic bioreactor treating paper mill wastewater (Pettine et al., 1999) (see **Figure 1A** and **Table 1**). The *arxA* gene of strain TC-1 was successfully amplified and was closely related (sequence identity: 99%) to those recovered from Tukh Lake (represented by HJ1A27 in **Figure 1B**). The morphology of *Desulfotomaculum* TC-1 cells was rod-shaped with rounded ends, 0.8–1.5 μm in length and 0.2–0.4 μm in width (**Supplementary Figure S2**). The optimum growth temperature and pH for TC-1 were 60°C and 6.8, respectively. The optimum growth temperature was consistent with the environmental condition of the spring where the TC-1 strain was isolated (57°C). Under optimum conditions, the doubling time of strain TC-1 was approximately 30 h. Strain TC-1 cannot grow on As(III) in the DSMZ 63 medium (without lactate).

**Table 1:** Physiological, phylogenetic and phenotypic comparisons between strain TC-1 and known *arxA* gene-containing strains.

Strain name	Temperature (°C)	Species	Isolation source	Reference
*Desulfotomaculum* TC-1	*60*	*Firmicutes*	A hot spring of Tengchong, China	The present study
*Alkalilimnicola ehrlichii* str. MLHE-1	*20*	*Proteobacteria*	Water column of Mono Lake, CA, United States	Oremland et al., 2002; Zargar et al., 2010, 2012
*Ectothiorhodospira* strain PHS-1	*43*	*Proteobacteria*	A hot spring in Paoha Island of Mono Lake, CA, United States	Kulp et al., 2008
*Halorhodospira halophila* SL1	*20*	*Proteobacteria*	Summer Lake, OR, United States	Challacombe et al., 2013
*Halomonas* sp. ANAO-440	*20*	*Proteobacteria*	An alkaline saline lake in Mongolia	Hamamura et al., 2014


### Variations of Total Arsenic, Arsenite and Arsenate during Arsenite Oxidation by Strain TC-1

**FIGURE 2:**
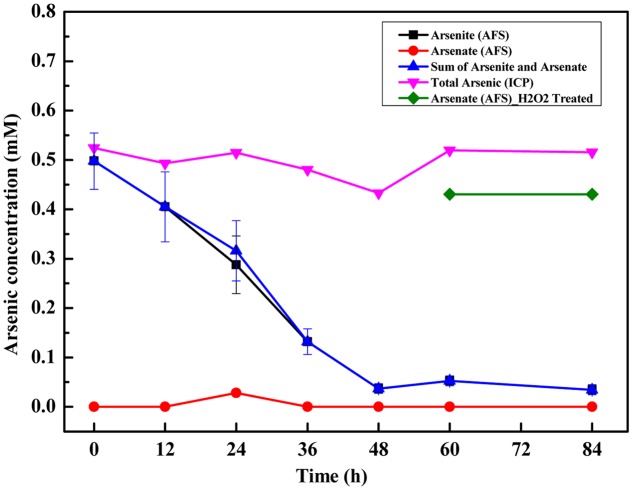
Time-course variations of arsenite and arsenate concentrations and total arsenic concentration during anaerobic arsenite oxidation by strain TC-1. Triplicate samples were performed, and error bars were smaller than the sizes of the symbols.

Arsenic speciation was examined for 84 h after inoculation with strain TC-1 (**Figure 2**). Arsenite (0.5 mM) was nearly exhausted in 60 h. As arsenite was consumed, some amount of arsenate was initially detected after 24 h (**Figure 2**). After 60 h, both arsenite and arsenate were not detectable in the experimental tubes, but the total arsenic in the solution remained unchanged, indicating no formation of insoluble arsenic precipitates. In the abiotic control containing Na_2_S and arsenite, white flocs formed in solution immediately. The resulting white flocs were separated by high speed centrifugation (12,000 rpm), and then were observed by SEM-EDS for element mapping. The results showed that arsenite could react with Na_2_S to form the flocs which contained S and As (**Supplementary Figure S3**). The resulted supernatant was treated with H_2_O_2_ followed by ICP-AES measurement, but no arsenic was detected (data not shown).

### Variation of Thioarsenic Species Formed during Arsenite Oxidation by Strain TC-1

**FIGURE 3:**
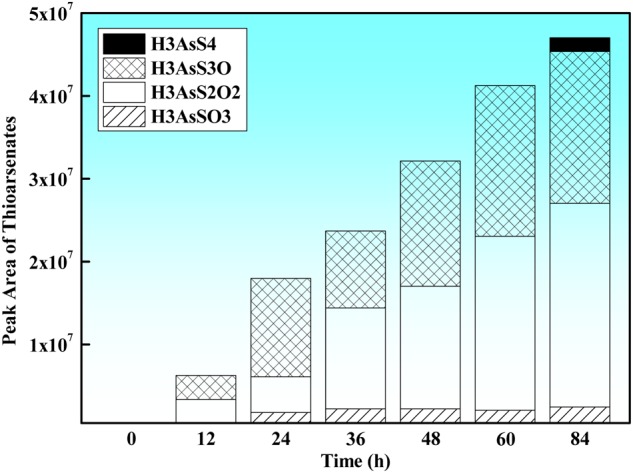
Time-course variation of thioarsenate composition (expressed as the corresponding chromatographic peak area of each identified thioarsenic species) produced during anaerobic arsenite oxidation by strain TC-1.

The LC-HRMS analysis showed that the missing arsenite had been transformed to thioarsenic species (mono-thioarsenate [HAsSO_3_]^2-^, di-thioarsenate [HAs^V^S_2_O_2_]^2-^, tri-thioarsenate [HAsS_3_O]^2-^ and tetra-thioarsenate [HAsS_4_]^2-^) (**Figure 3**), and the relative concentrations of each identified thioarsenic species varied during the oxidation process: In the first 60 h, monothioarsenate, di-thioarsenate and tri-thioarsenate were dominant species in the solution, while no tetra-thioarsenate was detected. As arsenite oxidation experiment proceeded, a small amount of tetra-thioarsenate was detected at 84 h (at the end of the experiment) (**Figure 3**). In the abiotic control containing Na_2_S and arsenate, only a tiny amount of di-thioarsenate was observed, but other thioarsenic species (i.e., monothioarsenate, tri-thioarsenate, and tetra-thioarsenate) were not detected.

## Discussion

### Anaerobic Arsenite Oxidation in Hot Springs

High levels of arsenic has been extensively reported in global terrestrial hot springs. Thus, terrestrial hot springs are an excellent setting for investigating arsenic biogeochemical cycling (Qin et al., 2009). In geothermal environments, microbially mediated aerobic arsenite oxidation has been frequently reported (Oremland, 2003), while few studies up to date discovered anaerobic arsenite oxidation by microbes. For example, Rhine et al. (2006) reported anaerobic arsenite oxidation phenomenon by novel denitrifying isolates from an arsenic contaminated industrial soil, while no function genes related to anaerobic arsenite oxidation were amplified; Zhao et al. (2015) reported anaerobic arsenite oxidation by an autotrophic arsenite-oxidizing bacterium from an arsenic-contaminated paddy soil and *aioA* gene was successfully amplified from those pure cultures, although the *aioA* gene is putatively involved in aerobic arsenic oxidation (Lett et al., 2012). Zargar et al. (2010) identified a novel arsenite oxidase gene, *arxA*, from *Alkalilimnicola ehrlichii* strain MLHE-1, an anaerobic arsenite-oxidizing bacterium from Mono Lake. However, little is reported on anaerobic arsenite oxidation by microbes in geothermal features. To our best knowledge, the only case of microbially mediated anaerobic arsenite oxidation in hot springs was reported in a hot spring (temperature 43°C) biofilm on the shore of the Paoha Island in Mono Lake (Kulp et al., 2008), which showed anaerobic photosynthetic arsenic(III) oxidation by strain *Ectothiorhodospira* strain PHS-1. However, in the present study, strain TC-1 can anaerobically oxidize arsenite at high temperature (60°C) independent of light or photosynthesis, indicating that photosynthesis-independent, microbially mediated anaerobic arsenic oxidation could take place in geothermal features.

To our best knowledge, strain TC-1 is the first known sulfate-reducing strain containing *arxA* gene. To date, only four other known *arxA* gene-containing strains have been obtained in pure cultures (**Table 1**) and they all fall within α*-Proteobacteria*, among which *A. ehrlichii* MLHE-1, *Halorhodospira halophila* SL1, and *Halomonas* sp. ANAO-440 were isolated from alkaline and/or saline environments, while *Ectothiorhodospira* strain PHS-1 was retrieved from a geothermal feature (temperature 43°C) (Oremland et al., 2002; Kulp et al., 2008; Zargar et al., 2010, 2012; Challacombe et al., 2013; Hamamura et al., 2014; Hernandez-Maldonado et al., 2016). In contrast, strain TC-1 belongs to *Firmicutes* and is the only known strain within *Firmicutes* possessing the *arxA* gene. The affiliation of strain TC-1 with *Firmicutes* indicated that the microbes involved in anaerobic arsenic oxidation may be more phylogenetically diverse than currently known. It is possible that the *arxA* gene of strain TC-1 might have originated from other microbes via horizontal gene transfer as in strain PHS-1 (Zargar et al., 2012).

### Formation of Thioarsenate under Anaerobic Conditions in Hot Springs

It is notable that thioarsenates could be formed by sulfate reducing bacteria under anaerobic conditions in hot springs. Previous work on arsenic speciation in geothermal environments reported the dominance of As(III) and As(V) in the bulk arsenic speciation (Ballantyne and Moore, 1988; Yokoyama et al., 1993; Macur et al., 2004). While studies with improved sample preservation techniques revealed that thioarsenate species were present or even abundant in geothermal features (Wilkin et al., 2003; Stauder et al., 2005; Planer-Friedrich et al., 2007; Wallschläger and Stadey, 2007; Guo et al., 2017), which can possibly make up to more than 50% of total dissolved arsenic in sulfidic waters (Wilkin et al., 2003; Hug et al., 2014). Most (if not all) of the geothermal features with reported high thioarsenates were sulfidic. The potential underlying reason for the formation of thioarsenates could be explained by the following equation:

$${\left[{HAs}^{V}O_{4}\right]}^{2-}+{\left[HS\right]}^{-}\to {\left[{HAs}^{V}{S^{-II}}_{x}O_{4-x}\right]}^{2-}+{\left[OH\right]}^{-},$$

in which arsenate reacted with sulfide, leading to the formation of thioarsenate (Planer-Friedrich et al., 2015). The reactant arsenate could be extant or derived from arsenite oxidation. Commonly anoxic condition dominates sulfidic habitats, thus anaerobic arsenite oxidation could take place to produce arsenate. In the present study, strain TC-1 could oxidize As(III) to As(V), which reacted with S^2-^ or HS^-^ and thus formed thioarsenate species. This reaction could also explain the wide distribution of thioarsenates in sulfidic aquifers (Wood et al., 2002; Wilkin et al., 2003; Bostick et al., 2005; Hollibaugh et al., 2005; Stauder et al., 2005; Wallschläger and Stadey, 2007), although no exact reasons were provided for the predominance of thioarsenates in those previous studies. The present study provides evidence for possible microbial involvement in the formation of thioarsenates in hot springs.

### Environmental Implication of Thioarsenate Formed by Sulfate Reducing Bacteria

Thioarsenate may be an important arsenic species in sulfidic and arsenic-rich environments (Hollibaugh et al., 2005; Hug et al., 2014; Guo et al., 2017). Based on the results presented above, high arsenic geological settings (e.g., groundwater and acid mine drainage that have the potential of sulfate reducing process) may contain significant amounts of thioarsenates, which to date have received little attention. Thioarsenates are more toxic than arsenate and tri-thioarsenate is almost as bioavailable and toxic as arsenite (Hinrichsen et al., 2015). Thus more attention should be paid to thioarsenates in high arsenic, sulfidic habitats. Previous studies have shown that arsenic in solution could be removed through combination with sulfide minerals derived from microbial SO_4_^2-^ reduction (Moore et al., 1988; Rittle et al., 1995; Kirk et al., 2004), and that the enhanced SO_4_^2-^ reduction may be useful for arsenic remediation (Rittle et al., 1995; Newman et al., 1997; Castro et al., 1999; Macy et al., 2000; Jong and Parry, 2003; Lee et al., 2005; Saunders et al., 2005; Keimowitz et al., 2007; Saunders et al., 2008; Kirk et al., 2010; Luo et al., 2013). However, recently an experiment with permeable reactive barriers (PRB) was performed to test the effect of arsenic remediation in the presence microbial sulfate reduction, and found that up to 47% of total As initially present in the sediment was leached out in the form of mobile thio-As species (Kumar et al., 2016). Thus, more cautions should be taken on the geochemical behaviors of arsenic and sulfate in the environment (where the arsenite and arsenate have the potential to transform to mobile thioarsenates) when sulfate reducing bacteria are employed for arsenic remediation (Burton et al., 2014; Stucker et al., 2014).

## Author Contributions

GW and HJ conceived and designed the experiments. LH and ZC isolated the strain. GW, YP, WG, HD, WS, and QG performed the experiments. GW analyzed the data. All of the authors assisted in writing the manuscript, discussed the results and commented on the manuscript.

## Conflict of Interest Statement

The authors declare that the research was conducted in the absence of any commercial or financial relationships that could be construed as a potential conflict of interest.

## Supplementary Material

**FIGURE S1 |** The location **(A)** and geothermal features **(B)** of DGG-2 in the Rehai Geothermal Field, Tengchong, Yunnan, China.

**FIGURE S2 |** Scanning electron microscope images of strain *Desulfotomaculum* sp. TC-1 cells (Scale bar = 1 μm).

**FIGURE S3 |** Scanning electron microscope images of flocs and the S-As distribution in floc as indicated by the yellow line in **(A)**. **(A–C)** Indicate the SEM morphology of the flocs and the EDX multi element mapping/line scanning spectra for As (arsenic) and S (sulfur) elements, respectively.
