# Supplementary figures and images for: Thioarsenate Formation Coupled with Anaerobic Arsenite Oxidation by a Sulfate-Reducing Bacterium Isolated from a Hot Spring
Source: Front Microbiol. 2017 Jul 14;8:1336. doi: 10.3389/fmicb.2017.01336 (PMC5509915; doi:10.3389/fmicb.2017.01336)

Fig S1

Fig. S2

Fig S3


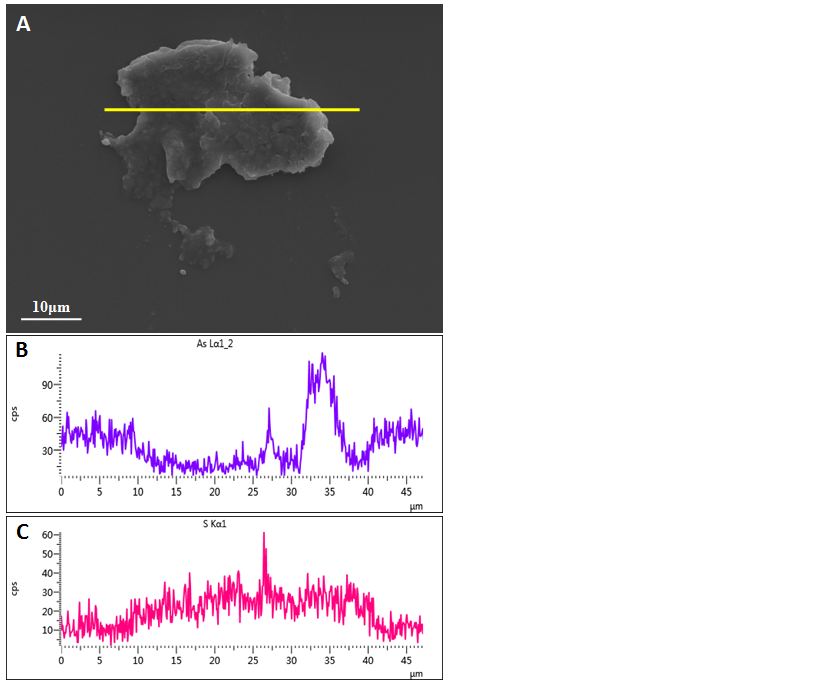

Supplement: Supplementary file 1 [file Data_Sheet_1.docx]
